# Additive Pressures of Elevated Sea Surface Temperatures and Herbicides on Symbiont-Bearing Foraminifera

**DOI:** 10.1371/journal.pone.0033900

**Published:** 2012-03-16

**Authors:** Joost W. van Dam, Andrew P. Negri, Jochen F. Mueller, Rolf Altenburger, Sven Uthicke

**Affiliations:** 1 The University of Queensland, School of Biological Sciences, St. Lucia, Australia; 2 Australian Institute of Marine Science, Townsville, Australia; 3 The University of Queensland, National Research Centre for Environmental Toxicology, Coopers Plains, Australia; 4 Helmholtz Centre for Environmental Research - UFZ, Dept. Bioanalytical Ecotoxicology, Leipzig, Germany; University of Sydney, Australia

## Abstract

Elevated ocean temperatures and agrochemical pollution individually threaten inshore coral reefs, but these pressures are likely to occur simultaneously. Experiments were conducted to evaluate the combined effects of elevated temperature and the photosystem II (PSII) inhibiting herbicide diuron on several types of symbiotic algae (diatom, dinoflagellate or rhodophyte) of benthic foraminifera *in hospite*. Diuron was shown to evoke a direct effect on photosynthetic efficiency (reduced effective PSII quantum yield *ΔF*/*F′_m_*), while elevated temperatures (>30°C, only 2°C above current average summer temperatures) were observed to impact photosynthesis more indirectly by causing reductions in maximum PSII quantum yield (*F_v_*/*F_m_*), interpreted as photodamage. Additionally, elevated temperatures were shown to cause bleaching through loss of chlorophyll *a* in foraminifera hosting either diatoms or dinoflagellates. A significant linear correlation was found between reduced *F_v_*/*F_m_* and loss of chlorophyll *a*. In most cases, symbionts within foraminifera proved more sensitive to thermal stress in the presence of diuron (≥1 µg L^−1^). The mixture toxicity model of Independent Action (IA) described the combined effects of temperature and diuron on the photosystem of species hosting diatoms or dinoflagellates convincingly and in agreement with probabilistic statistics, so a response additive joint action can be assumed. We thus demonstrate that improving water quality can improve resilience of symbiotic phototrophs to projected increases in ocean temperatures. As IA described the observed combined effects from elevated temperature and diuron stress it may therefore be employed for prediction of untested mixtures and for assessing the efficacy of management measures.

## Introduction

A dramatic decline in coral cover has been recorded in the last three decades [1], primarily driven by an increasing frequency of climate-related mass mortality events [2], [3]. Predicted increases in the frequency and duration of high summer temperatures [4] exceeding species' thermal tolerance thresholds present a significant risk to the biodiversity of coral reefs and to the services they provide [5], [6]. It has been implied that the earliest symptoms of heat damage in reef-building organisms are associated with limitation of photosynthetic electron flow [7]–[11] and carboxylation within the Calvin cycle of symbiotic microalgae [12], [13]. Excess excitation energy that cannot be utilized in photochemical charge separation subsequently overwhelms photoprotective mechanisms, leading to oxidative stress and photoinhibition [14]. In corals and other symbiotic reef species, this can cause loss of symbiotic algae or reduced pigment concentrations (bleaching).

Pollution from terrestrial runoff also negatively affects reef health [15]. In the last century and a half, intensive agriculture and industries along the Queensland coastline have significantly increased the annual input of suspended sediments and nutrients into the Great Barrier Reef (GBR) lagoon [16]. Correspondingly, the use of pesticides in catchments that flow into the GBR has been growing steadily [17]. Recent studies have found contemporary herbicides to be ubiquitous in nearshore areas of the GBR [18]–[20]. Photosystem II (PSII) herbicides are of particular ecological concern with regards to reef systems, as they are relatively mobile and safety margins between chronic environmental concentrations and effect concentrations as determined by laboratory studies are relatively small [21]. These compounds act by inhibiting electron transport through the photosystem in chloroplasts by reversibly binding to a specific electron-acceptor protein (D1-enzyme) in PSII, outcompeting the normal ligand for binding sites [22]. Intracellular microalgae of symbiotic reef species are likewise affected by herbicides and will suffer reduced photosynthetic efficiency, limiting energy flow from symbiont to host [23]. High inhibitor concentrations or sustained blockage of the electron transport chain can cause secondary effects through the reduced availability of ATP and NADPH and the formation of reactive oxygen species, causing chronic photoinhibition [24].

Inhibition of photosynthesis in symbiotic organisms can decrease production and leads to photosystem damage and in turn bleaching, thus disturbing the fragile relationship so important in coral reef ecology. However, not all symbiont bearing organisms display equal vulnerability to environmentally adverse conditions. For example, genetic diversity within dinoflagellates of the genus *Symbiodinium* that complement the host-symbiont relationship in corals largely influences holobiont resilience to environmental stress such as higher than usual temperatures [25], [26] or pollution [23], allowing for ecological adaptation. While the majority of stress-response studies have been performed on corals, other species may also be at risk. Foraminifera are single-celled protists that may form symbiotic relationships with several different microalgal phyla, including diatoms, dinoflagellates, red and green algae [27]. Foraminifera are widespread, sensitive to environmental change [28], [29] and have been documented to bleach under stressful conditions in the field [30]–[34]. Furthermore, they can be employed as indicator species for water quality assessment [35], [36]. A wide suit of laboratory studies have demonstrated reduced growth and/or bleaching at elevated temperatures, UV or increased irradiance, elevated nutrient levels or combinations of stressors for several species [37]–[41]. Recently, we demonstrated how several different species of benthic foraminifera, hosting four different microalgal phyla, exhibit widely varying responses to the PSII herbicide diuron [42], while another recent study has linked changes in foraminiferal community structure to an increase in terrestrial runoff [43].

In the tropics, summer monsoonal rainfall and subsequent river flooding occur when sea surface temperatures (SSTs) approach tolerance threshold levels for many species, thereby simultaneously exposing inshore reefs to combinations of low salinity, high turbidity, nutrients and agrochemical residues during episodes of thermal stress. Despite this, water quality guidelines are based on known thresholds and impacts of single stressors, reflecting the majority of stress-response studies, while environmentally important combinations of stressors are rarely considered. Regulatory agencies have recently recognized the potential for pollution to reduce the resilience of reef systems and have adopted strategies of optimizing water quality in order to protect sensitive species to the effects of global climate change [44], [45]. However, empirical support for this strategy is limited and heavily relies on results obtained from studies on hard corals [46]–[50].

Increased temperatures may cause conformational changes in the D1-protein and so change a herbicide's binding affinity [51]. Furthermore, as in corals, foraminiferal symbionts reside within their host cells [27] and thus the herbicide will have to cross several membrane layers (of both host and algal origin) to reach its target site on the D1-protein [52]. Temperature affects membrane permeability and internal cellular processes such as protein repair mechanisms or bio-elimination and may therefore enhance or reduce toxicity of pollutants [53]. Since thermal stress and herbicides both target symbiont photochemistry, additive or interactive effects may occur, as has been recently shown in corals [46]. The aims of the present study were to test how the susceptibility (thresholds) of various symbiotic partnerships (benthic foraminifera and their intracellular microalgae) to the adverse effects of elevated SSTs changes in the presence of the PSII herbicide diuron.

## Materials and Methods

All experiments were undertaken at the Australian Institute of Marine Science (AIMS), Townsville, Australia. Benthic foraminifera were collected from two sites along the GBR (Orpheus Island and Lizard Island) between February 2009 and April 2010. All necessary permits were obtained prior to field collections. Species were separated and kept at 26°C in 500 mL plastic beakers containing 0.5 µm filtered seawater (FSW), refreshed every second day. Maintenance and dosage experiments were conducted under a 12 h∶ 12 h diurnal light-dark cycle using 10,000K compact fluorescent globes. Irradiance intensity was set at 10 µmol quanta m^−2^s^−1^ PAR, considered suitable for all species tested here as determined previously [42], [54]. Foraminifera used in the bioassays are listed in Table 1.

**Table 1:** Foraminifera used in this study, symbiont type and collection data.

Species	Symbiont type	Location collected	Depth collected
*Heterostegina depressa*	Diatoms	18°39′4.9″S–146°29′10.2″E	7–9 m
*Calcarina mayorii*	Diatoms	18°39′4.9″S–146°29′10.2″E	3–7 m
*Alveolinella quoyi*	Diatoms	18°39′4.9″S–146°29′10.2″E	7–9 m
*Marginopora vertebralis* (inshore)	Dinoflagellates	18°39′4.9″S–146°29′10.2″E	2–4 m
*Marginopora vertebralis* (offshore)	Dinoflagellates	14°38′59.2″S–145°27′38.5″E	2–3 m
*Peneroplis planatus*	Rhodophytes	18°39′4.9″S–146°29′10.2″E	7–9 m

### 96-hour co-exposures

Foraminifera were exposed to low concentrations of diuron and tested against a solvent control (carrier only) at temperatures of 26°C, 28°C, 30°C, 32°C or 34°C (±0.2°C) in a set of 5 high-precision incubators. These temperatures represent a range from the mean annual temperature of seawater from inshore reefs off Townsville to a high estimation of expected temperatures under projected climate change scenarios for the coming century. Two days prior to diuron exposure, specimens were placed in the 26°C incubator for a 24 hour adaptation period, after which specimens were transferred to polystyrene tissue culture plates with wells containing 10 mL FSW. *Heterostegina depressa* and *Marginopora vertebralis* were individually placed in a well, while for the other (smaller) species tested, several specimens were pooled. Experiments were set up in a full orthogonal design with 6 replicate wells used per exposure combination. Plates were set up fully randomized, heated over a 24 hour time period (max. increase 0.33°C per hour) and kept at experimental temperatures for 6 hours before FSW was refreshed and diuron introduced. Analytical grade diuron (Sigma-Aldrich) was used to daily prepare fresh stock solutions in FSW with DMSO as carrier (final concentrations in experimental media <0.05% (v/v)). Exposure media were changed daily to obtain final nominal concentrations of 0, 1 or 3 µg L^−1^ diuron in 10 mL FSW. Riedl and Altenburger [55] recommend exposure concentrations be measured for compounds with high hydrophobicities (log K*ow*>3) as toxicant adsorption to test vessels is expected at log K*ow*>4 [56]. Diuron is not volatile and of moderate hydrophobicity with a log K*ow* of 2.6 [57], therefore absorption was unlikely and nominal concentrations deemed appropriate. Prior to the initial introduction of diuron and for every 24 hours thereafter until the end of the experiment after 96 hours exposure, both effective (*ΔF*/*F′_m_*) and maximum quantum PSII yields (*F_v_*/*F_m_*) were determined for assessment of photosynthetic performance (see following section for details). At the end of the experiment specimens were snap frozen in liquid nitrogen and transferred to a −80°C freezer in preparation for pigment extraction.

### Chlorophyll fluorescence techniques

The saturation pulse method [58] using a MINI-PAM fluorometer (Walz GmbH, Germany) was applied following van Dam *et al.* [42] to evaluate stressor effects on photochemical pathways of symbiotic algae *in hospite*. A weak pulse-modulated red measuring light (650 nm, 0.15 µmol quanta m^−2^s^−1^ PAR) was applied to the sample to measure baseline fluorescence (*F* in experimental light conditions or *F_0_* after dark adaption), followed by a short (800 ms) but saturating light pulse (>3000 µmol quanta m^−2^s^−1^ PAR) to measure maximal fluorescence (*F′_m_* in illuminated samples or *F_m_* in a dark adapted sample). The quantum yield of PSII can be calculated following Genty *et al.* [59]:

(*F′_m_−F*)/*F′_m_* = *ΔF*/*F′_m_* for the effective quantum PSII yield in an illuminated sample; and

(*F_m_−F_0_*)/*F_m_* = *F_v_*/*F_m_* for the maximum potential quantum PSII yield in a dark adapted sample.

*ΔF*/*F′_m_* is a measure of ‘open’ reaction centres and directly proportional to energy conversion in PSII [60]. *F_v_*/*F_m_* indicates potential energy conversion at PSII and reductions in *F_v_*/*F_m_* signify damage to PSII [61].

### Bleaching assessment

Evaluation of chlorophyll *a* (Chl *a*) content is often used as a proxy for bleaching (loss of pigments or algal cells) in studies on coral stress. We quantified Chl *a* in *H. depressa* and *M. vertebralis* (Table 1) by spectrophotometry following the temperature-herbicide treatments. Pigments were extracted in 95% ethanol following van Dam *et al.* [42], absorbance was measured at 665 and 750 nm using a Synergy HT plate reader (Bio-Tek) and Chl *a* calculated as per Schmidt *et al.* [62].

### Data analysis

The evaluation of the combined effects of simultaneous stressors is usually based upon one of two different reference concepts: Concentration Addition (CA) or Independent Action (IA - often referred to as response addition) [63]. Both models are widely used in pharmacology and ecotoxicology and predict combined additive effects from the toxicity of the individual components; CA is said to occur when stressors have similar target sites and IA applies when molecular mechanisms are different [64]. As stressors used here are likely to have independent modes of action, predicted additive inhibition of PSII yields *ΔF*/*F′_m_* and *F_v_*/*F_m_* was calculated across the experimental range according to the reference model of IA [65]:
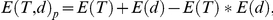
Where *E*(*T,d*)*_p_* is the predicted combined effect in case of response additivity; *E*(*T*) is the effect of temperature in the absence of diuron and *E*(*d*) is the effect of diuron at the control temperature of 26°C, both derived from raw data means. Next, the observed (measured) combined effect of diuron and temperature on PSII yield *E*(*T,d*)*_o_* was plotted against *E*(*T,d*)*_p_*. Raw data means and their 95% confidence intervals (for observed data) overlapping the zero-interaction line indicate response additivity, while datapoints above or below the line indicate sub-additivity (combined response smaller than predicted) or synergism (combined response greater than predicted).

Analysis of variance (ANOVA) was applied to analyze individual and combined effects of elevated temperature and diuron on response parameters. First, yield and Chl *a* data were tested for normality of distribution (Kolmogorov D-test) and homogeneity of variance (modified Levene's test) to meet test assumptions. Data not satisfying criteria were arcsine transformed; in case heterogeneity of variance persisted, rank-transformed data were used. Data meeting all assumptions were compared across treatments using two-way factorial ANOVA (*α*<0.050) with temperature and concentration as fixed factors. Tukey-Kramer's post-hoc test was used to identify treatment groups significantly different from other treatment groups. To control for Type I errors, *α* was adjusted for the number of separate post-hoc tests [66]. These analyses were performed using NCSS 2007 (statistical software package).

In order to quantify how the PSII-inhibiting effect of temperature changed with increasing diuron concentrations, a two-predictor polynomial curve for combined effects (linking temperature and diuron concentration) was fitted to each dataset using linear models in the R statistical platform [67]:
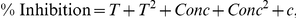
Where *T* is temperature (°C); *Conc* is diuron concentration (µg L^−1^) and *c* represents a constant. Inclusion of a second order polynomial term for concentration (*Conc^2^*) did not improve fit for any of the models (ANOVA test for model comparison) and was excluded from the model. As minimal effect levels were examined, effective temperatures eliciting 10 and 25% inhibition of photosynthetic yield (IT_10_ and IT_25_, respectively) at chosen diuron concentrations were calculated and reported.

## Results

### Dose-dependent functional effects on ΔF/F′_m_

**Figure 1:**
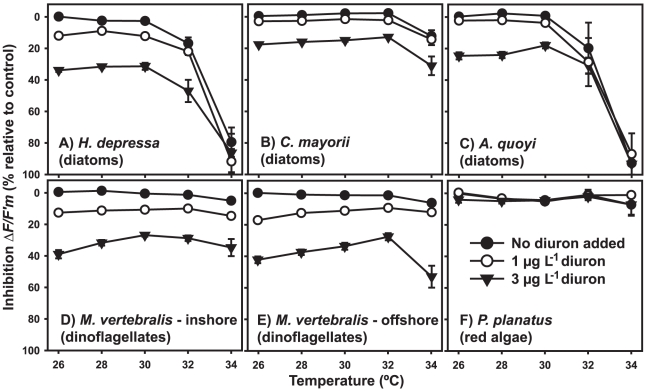
Relative inhibition of effective quantum PSII yield (*ΔF*/*F′_m_*) of symbiotic microalgae of six species of benthic foraminifera. *ΔF*/*F′_m_* was measured *in hospite*, following 96 h exposure to low diuron concentrations at temperatures ranging from 26–34°C. Data are means (*x*) ± SE, n = 6 specimens per treatment.

Combined effects of temperature and diuron were tested in bioassays for 6 species of foraminifera (Table 1). Both temperature and diuron had a significant effect on effective quantum PSII yield *ΔF/F′_m_* for all species except for the red algae bearing *P. planatus* (Table 2). The diuron-induced inhibition of *ΔF*/*F′_m_* remained relatively constant up to 30°C, with up to 12% inhibition caused by 1 µg L^−1^ diuron in diatoms (*H. depressa* 6–12%, *C. mayorii* 2–4.5%, *A. quoyi* 2.5–4.5%) and 10–17% inhibition in dinoflagellates (Fig. 1A–E). At 3 µg L^−1^ diuron, up to 34% inhibition of *ΔF*/*F′_m_* in diatoms (*H. depressa* 30–34%, *C. mayorii* 17%, *A. quoyi* 18–27%, Fig. 1A–C) was observed, while 26–39% inhibition was observed in dinoflagellates up to 30°C (Fig. 1D and 1E).

**Table 2:** Summary of two-factor ANOVA of *ΔF*/*F′_m_* and *F_v_*/*F_m_* of different foraminiferal symbionts, measured *in hospite*, in response to 96 h exposures to combinations of elevated temperature and diuron.

Species	Parameter	Factor	df	MS	*F*	*p*
*H. depressa* a	*ΔF*/*F′_m_*	T	3	0.028575	26.62	0.000000*
		Conc	2	0.265557	247.36	0.000000*
		T*Conc	6	0.001698	1.58	0.168447
		Residuals	59	0.001074		
	*F_v_*/*F_m_*	T	3	0.034245	44.93	0.000000*
		Conc	2	0.019606	25.72	0.000000*
		T*Conc	6	0.000349	0.46	0.836894
		Residuals	58	0.000762		
*C. mayorii*	*ΔF*/*F′_m_*	T	4	0.051743	29.09	0.000000*
		Conc	2	0.193762	108.94	0.000000*
		T*Conc	8	0.000373	0.21	0.988349
		Residuals	75	0.001779		
	*F_v_*/*F_m_*	T	4	0.03804	28.96	0.000000*
		Conc	2	0.031098	23.67	0.000000*
		T*Conc	8	0.000442	0.34	0.949221
		Residuals	75	0.001314		
*A. quoyi* a	*ΔF*/*F′_m_*	T	3	0.082038	5.7	0.001679*
		Conc	2	0.142142	9.88	0.000196*
		T*Conc	6	0.007794	0.54	0.774535
		Residuals	60	0.014393		
	*F_v_*/*F_m_*	T	3	0.10161	6.97	0.000421*
		Conc	2	0.010673	0.73	0.485011
		T*Conc	6	0.007357	0.5	0.802251
		Residuals	60	0.014573		
*M. vertebralis*	*ΔF*/*F′_m_*	T	4	342.0993	10.38	0.000001*
(inshore)		Conc	2	24790.17	751.84	0.000000*
		T*Conc	8	282.0422	8.55	0.000000*
		Residuals	73	32.9726		
	*F_v_*/*F_m_*	T	4	0.017098	58.71	0.000000*
		Conc	2	0.043917	150.81	0.000000*
		T*Conc	8	0.000381	1.31	0.252717
		Residuals	74	0.000291		
*M. vertebralis*	*ΔF*/*F′_m_*	T	4	1538.906	8.58	0.000004*
(offshore)		Conc	2	73598.07	410.48	0.000000*
		T*Conc	8	841.2081	4.69	0.000054*
		Residuals	115	179.2957		
	*F_v_*/*F_m_*	T	4	5363.344	9.45	0.000001*
		Conc	2	42444.93	74.79	0.000000*
		T*Conc	8	533.4131	0.94	0.486796
		Residuals	114	567.5298		
*P. planatus*	*ΔF*/*F′_m_*	T	4	0.001611	1.46	0.22433
		Conc	2	0.001322	1.2	0.308141
		T*Conc	8	0.000437	0.4	0.919174
		Residuals	69	0.001104		
	*F_v_*/*F_m_*	T	4	0.001042	0.87	0.487574
		Conc	2	0.001866	1.56	0.218489
		T*Conc	8	0.001173	0.98	0.460818
		Residuals	69	0.0012		

a Data incorporated in the analysis limited to 26–32°C treatments as excessive mortality occurred in the 34°C treatments.

* Significant factor (*α* = 0.050 or adjusted for # post-hoc tests). Some photosynthetic yield data were arcsine or rank transformed prior to analysis. T = temperature, Conc = diuron concentration.

The negative effect of elevated temperature became evident in treatments ≥32°C in diatom-bearing species. The species hosting diatoms that had been collected at slightly greater depth (*H. depressa* and *A. quoyi*, Table 1), were most severely affected with a significant temperature-induced decrease in *ΔF*/*F′_m_* occurring over 30°C (17–20% inhibition occurring at 32°C, *p*<0.004 (adjusted post-hoc *α*)). For these species, 96 hours incubation at 34°C proved lethal. *Calcarina mayorii*, a diatom bearer that also exists on the reef flat, and the shallow-dwelling, dinoflagellate-hosting *M. vertebralis* proved less sensitive to high temperatures with up to 12% inhibition of *ΔF*/*F′_m_* observed at 34°C (*C. mayorii* 12% - Fig. 1B, *M. vertebralis* 5–12% - Fig. 1D and 1E).

A linear curve fit linking temperature and diuron concentration in a single 2-predictor data model allowed for a crude quantification of the diuron-dependent effect of elevated temperature on PSII. Calculated R^2^-values for modeled inhibition of *ΔF*/*F′_m_* ranged from 0.72 to 0.90, explaining a great part of the variance within the dataset (Table 3). The models derived were used to calculate temperatures at which 10 and 25% inhibition of photosynthetic yield occurred (IT_10_ and IT_25_, respectively) for the chosen diuron exposures and demonstrated that temperature-induced PSII inhibition always decreased in the presence of low concentrations of diuron, indicative of how diuron actively lowers temperature thresholds for inhibition of photosynthesis (Table 3). In *H. depressa* for example, the model predicted that reducing the diuron concentration from 2 to 0 µg L^−1^ at 31°C, will reduce temperature-induced inhibition of *ΔF*/*F′_m_* from 25% to 10%. Similarly, lowering the diuron concentration from 1 to 0.5 µg L^−1^ at 30°C will avert a similar inhibition of *ΔF*/*F′_m_* in *M. vertebralis* as would be caused by a temperature increase of 3°C. For all species, 96 hour IT_10_-values were at least 0.5°C higher in the absence of diuron when compared with 1 µg L^−1^ diuron (Table 3).

**Table 3:** Two-predictor polynomial curves describing how the PSII-inhibiting effect of elevated temperature changed with increasing diuron concentrations.

Response parameter	R^2^	Equation	IT_10_			IT_25_		
*Species*	(*p*<0.001)	(*Y* = % inhibition)	No diuron	1 µg L^−1^ diuron	3 µg L^−1^ diuron	No diuron	1 µg L^−1^ diuron	3 µg L^−1^ diuron
**Inhibition ** ***ΔF*** **/** ***F′_m_***								
*H. depressa*	0.807	*Y* = 1833−130*T+2.29*T^2^+8.57*Conc	30.7	29.8	-	31.8	31.3	29.4
*C. mayorii*	0.727	*Y* = 478−33.3*T+0.58*T^2^+5.92*Conc	34.2	33.1	-	36.3	35.6	33.8
*A. quoyi*	0.750	*Y* = 2157−153*T+2.70*T^2^+5.62*Conc	30.8	30.3	-	31.7	31.4	30.6
*M. vertebralis* (inshore)	0.893	*Y* = 280−18.8*T+0.32*T^2^+10.4*Conc	35.8	31.3	-	38.9	36.9	-
*M. vertebralis* (offshore)	0.818	*Y* = 426−28.8*T+0.48*T^2^+12.3*Conc	34.7	-	-	37.2	35.3	-
**Inhibition ** ***F_v_*** **/** ***F_m_***								
*H. depressa*	0.766	*Y* = 1853−132*T+2.34*T^2^+2.93*Conc	31	30.7	30.2	31.9	31.8	31.4
*C. mayorii*	0.570	*Y* = 365−25.6*T+0.44*T^2^+2.23*Conc	34.1	33.7	32.5	36.7	36.4	35.7
*A. quoyi*	0.766	*Y* = 2175−155*T+2.74*T^2^+1.58*Conc	30.5	30.3	30	31.5	31.4	31.2
*M. vertebralis* (inshore)	0.690	*Y* = 232−16.7*T+0.30*T^2^+3.82*Conc	33.5	32.2	-	36.9	36.1	34.4
*M. vertebralis* (offshore)	0.526	*Y* = 418−29.0*T+0.50*T^2^+4.78*Conc	34	33	-	36.4	35.8	34.2

Inclusion of a second order polynomial term for concentration (Conc^2^) did not improve fit for any of the models (ANOVA test for model comparison) and was excluded from the model. Equations linking temperature and diuron concentration were fitted to the inhibition data and solved to obtain temperatures where 10 and 25% inhibition *ΔF*/*F′_m_* and *F_v_*/*F_m_* occurred (IT_10_ and IT_25_, respectively). The adjusted R^2^ indicates which part of the variance in the dataset is explained by the model. Empty fields signify diuron concentrations at which >10 or 25% inhibition PSII yield was observed, irrespective of temperature. T = temperature (°C), Conc = diuron concentration (µg L^−1^).

**Figure 2:**
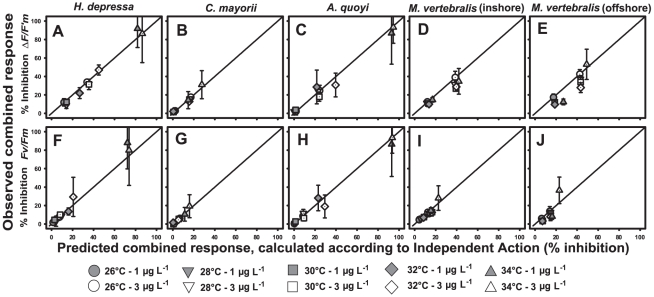
Correlation between observed and predicted (IA) combined effects of elevated temperature and diuron on photosynthesis of foraminiferal symbionts. Datapoints overlapping the zero-interaction line indicate additivity; datapoints underneath the additivity line indicate sub-additivity (combined effect<predicted effects); and datapoints above the additivity line indicate synergism (combined effect>predicted effects). **A–E**) Correlations for inhibition *ΔF*/*F′_m_* after 96 h exposure. **F–J**) Correlations for inhibition *F_v_*/*F_m_* after 96 h exposure. Data are means (*x*) ±95% CI, n = 6 specimens per treatment.

The mixture toxicity model of Independent Action (IA) was used to evaluate possible interactive effects of elevated temperature and diuron exposure. Plotting measured against predicted combined response (IA) (Fig. 2A–C) yielded near perfect agreement between observed and predicted effect for diatom-bearing species *H. depressa*, *C. mayorii* and *A. quoyi*, indicative of response additivity for this combination of stressors on these species. The inhibition graphs for both types of *M. vertebralis* (dinoflagellates) suggest a slight photoprotective effect for diuron-induced inhibition of *ΔF*/*F′_m_* at temperatures between 28–32°C when compared to 26 or 34°C (Fig. 1D and 1E). Indeed, here ANOVA yielded a significant interaction term (*p*<0.001, Table 2). For the inshore variety this interaction was assessed as sub-additive since the observed combined effects of temperature and diuron were less than predicted in case of response additivity (IA) (Fig. 2D). As for the specimens collected offshore, no consistent trend was observed (Figs. 1E and 2E). On the contrary; diuron-induced inhibition of *ΔF*/*F′_m_* was lowest at 32°C while at temperatures >32°C the diuron effect was much more pronounced (Fig. 1E). A comparison between the observed and predicted response (IA) revealed that in the intermediate temperature regions, the predicted combined response was greater than the observed combined response, where at higher and lower temperatures predicted and observed inhibition were equivalent (Fig. 2E).

### Dose-dependent functional effects on F_v_/F_m_

**Figure 3:**
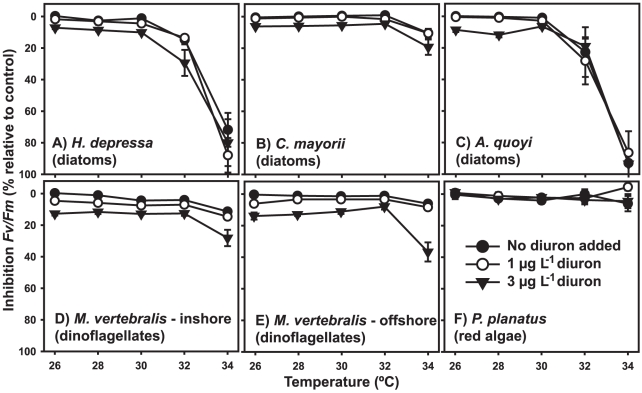
Relative inhibition of maximum quantum PSII yield (*F_v_*/*F_m_*) of symbiotic microalgae of six species of benthic foraminifera. *F_v_*/*F_m_* was measured in hospite, following 96 h exposure to low diuron concentrations at temperatures ranging from 26–34°C. Data are means (*x*) ± SE, n = 6 specimens per treatment.

Combined stressor inhibition curves for inhibition *F_v_*/*F_m_* (Fig. 3) followed similar patterns as those describing inhibition *ΔF*/*F′_m_* (Fig. 1). ANOVA revealed significant effects of both temperature and diuron on *F_v_*/*F_m_* for any of the species tested except for *A. quoyi* (unaffected by diuron) and *P. planatus* (unaffected by neither stressor) (Table 2). After 96 hours incubation, 1 µg L^−1^ diuron did not significantly decrease *F_v_*/*F_m_* in diatom-bearing species (Fig. 3A–C), but did so for *M. vertebralis* hosting dinoflagellates (4.5–6% reduction, *p*<0.003 (adjusted post-hoc *α*)) (Fig. 3D and 3E). 3 µg L^−1^ diuron caused 6–11% photodamage in diatoms and 7–17% in dinoflagellates (Table 2; Fig. 3).

Temperature-induced inhibition of *F_v_*/*F_m_* was equivalent to inhibition observed for *ΔF*/*F′_m_*. ‘Deep’ (>9 m) diatom-bearers were affected at temperatures >30°C [14% and 22% inhibition *F_v_*/*F_m_* for *H. depressa* for and *A. quoyi* at 32°C, respectively - Figure 3A and 3C, *p*<0.004 (adjusted post-hoc *α*)], while ‘shallow’ (<7 m) *C. mayorii* (Fig. 3B) and dinoflagellate-hosting *M. vertebralis* (Fig. 3D and 3E) were only significantly affected at temperatures >32°C (10–11% inhibition *F_v_*/*F_m_* at 34°C, *p*<0.003 (adjusted post-hoc *α*)).

Linear curve fits for inhibition *F_v_*/*F_m_* demonstrated how diuron lowered temperature thresholds for the onset of photodamage (Table 3). The total combined effect on *ΔF*/*F′_m_* was stronger than on *F_v_*/*F_m_*, which may be explained by the fact that *F_v_*/*F_m_* was not as greatly affected by diuron. However, despite the smaller effect, diuron effectively lowered IT_10_ and IT_25_ for inhibition *F_v_*/*F_m_* (Table 3). Again, a high agreement between measured and predicted (IA) combined effects on *F_v_*/*F_m_* indicated response additivity also for this parameter (Fig. 2F–J).

### Time-dependent functional effects on ΔF/F′_m_ and F_v_/F_m_

**Figure 4:**
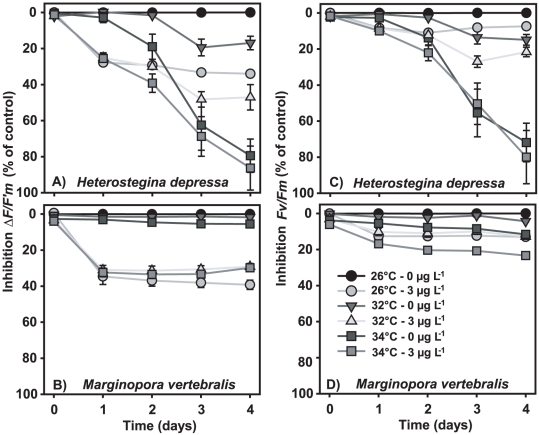
Quantum PSII yield inhibition kinetics of symbiotic microalgae of *Heterostegina depressa* and *Marginopora vertebralis*. Specimens were exposed to combinations of diuron and temperature over 96 h. **A+B**) inhibition kinetics *ΔF*/*F′_m_*. **C+D**) inhibition kinetics *F_v_*/*F_m_*. Data are means (*x*) ± SE, n = 6 specimens per treatment.

Inhibition of *ΔF*/*F′_m_* and *F_v_*/*F_m_* by temperature-herbicide combinations revealed characteristic patterns over time for species hosting different symbiont types. Time series plots for inhibition of PSII yield are presented for *H. depressa* and *M. vertebralis* in Figure 4. For both species, the effect of diuron was apparent from day 1, remaining relatively constant for the remainder of the experiment. Incubation in 3 µg L^−1^ caused ∼25% inhibition of *ΔF*/*F′_m_* in the diatom-bearing *H. depressa* (Fig. 4A) and ∼35% in the dinoflagellate-hosting *M. vertebralis* (Fig. 4B). Diuron-induced reduction of *F_v_*/*F_m_* was less apparent with ∼10% inhibition in *H. depressa* (Fig. 4C) and ∼10–20% in *M. vertebralis* (Fig. 4D). Temperature on the other hand did not immediately affect either response parameter. For *H. depressa* incubated at 34°C, ∼20% inhibition of *ΔF*/*F′_m_* manifested after 2 days exposure (Fig. 4A). At that time, ∼15% inhibition of *F_v_*/*F_m_* was evident (Fig. 4C). After 3 days exposure at 32°C, ∼20% and ∼15% inhibition of *ΔF*/*F′_m_* and *F_v_*/*F_m_* had occurred. The temperature-induced inhibition of *ΔF*/*F′_m_* and *F_v_*/*F_m_* increased over time, with 4 days incubation at 34°C causing complete functional inhibition in this species. In contrast, *M. vertebralis* was little affected after 96 hours by temperatures up to 34°C, with ∼10% maximum inhibition observed (Fig. 4B and 4D). As hypothesized from 96 hour inhibition results (Figs. 1 and 3), these data (Fig. 4) confirmed that diuron acutely inhibits *ΔF*/*F′_m_*, leading to a loss in photosynthetic efficiency. At low concentrations and for short exposure durations, diuron has relatively little impact on *F_v_*/*F_m_* (∼10% inhibition at 3 µg L^−1^). In contrast, high temperatures cause photodamage in sensitive species as indicated by a decrease in *F_v_*/*F_m_* and equivalent drop in *ΔF*/*F′_m_*. These effects only become apparent over time and may increase, depending on evolution of the temperature regime.

### Chlorophyll a content

**Figure 5:**
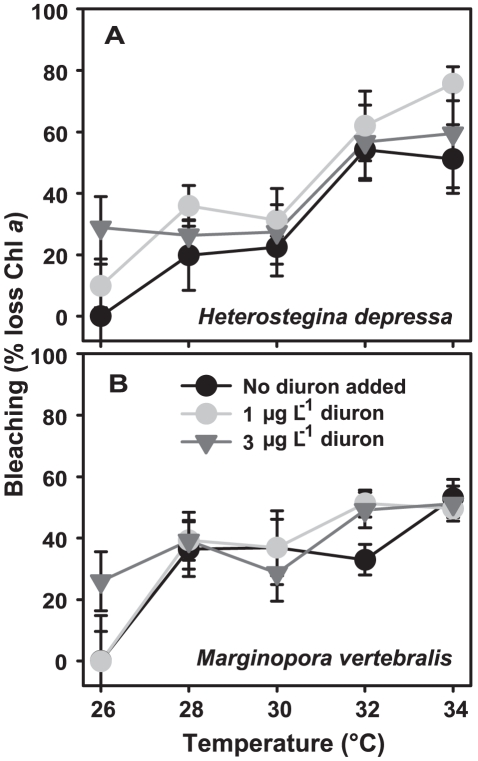
Combined effects of diuron and temperature on Chl *a* content of *Heterostegina depressa* and *Marginopora vertebralis*. Chl *a* content was determined after 96 h exposures and expressed relative to wet weight. Data are means (*x*) ± SE, n = 6 (*M. vertebralis*) or 4 (*H. depressa*) specimens per treatment.

Reduction in Chl *a* content was used as a proxy for bleaching in both *H. depressa* and *M. vertebralis* (Fig. 5). For both species, 96 hour exposure to diuron up to 3 µg L^−1^ had no significant effect on Chl *a* content (Table 4). Loss of Chl *a* was observed for both species exposed for 96 hours at temperatures between 28–30°C (∼10–40% in *H. depressa* and ∼30–50% in *M. vertebralis*), with more extensive bleaching occurring at higher temperatures (∼40–80% in *H. depressa* and ∼40–60% in *M. vertebralis*) (Fig. 5A and 5B; Table 4). No statistical interaction between pressures was revealed (Table 4). However, moderately significant linear relationships were detected between bleaching (loss of Chl *a*) and chronic photodamage (reduced *F_v_*/*F_m_*) in both *H. depressa* (R^2^ = 0.532, *p* = 0.020) and *M. vertebralis* (R^2^ = 0.300, *p* = 0.034) (data not shown).

**Table 4:** Summary of two-factor ANOVA of chlorophyll *a* content of *Heterostegina depressa* and *Marginopora vertebralis* after 96 hours exposure to combinations of diuron and temperature.

Species	factor	df	MS	*F*	*p*
*H. depressa*	T	3	10410.96	6.38	0.001509*
	Conc	2	1431.722	0.88	0.425130
	T*Conc	6	527.8601	0.32	0.920097
	Residuals	34	1632.066		
*M. vertebralis*	T	4	21961.86	11.05	0.000000*
	Conc	2	1674.28	0.84	0.434833
	T*Conc	8	2032.207	1.02	0.427077
	Residuals	75	1988.201		

* Significant factor (*α* = 0.050 or adjusted for # post-hoc tests). T = temperature, Conc = diuron concentration.

## Discussion

### Synthesis

This was the first investigation into combined effects of elevated temperature and low levels of pollution on the photochemistry of different symbiotic partnerships in various species of foraminifera. The negative effects of elevated temperatures on symbiont photochemistry were in most cases more severe in the presence of low concentrations of diuron, as might be expected since both factors are independently capable of impacting PSII. Diuron had a direct effect on photosynthetic efficiency, while elevated temperatures impacted photosynthesis indirectly by causing photodamage. These effective temperatures correspond with not unlikely predictions and may occur by the end of this century. Low concentrations of diuron were found to reduce temperature thresholds for inhibition of photosynthesis and, to a somewhat lesser extent, the onset of photodamage. Additionally, elevated temperatures were shown to cause bleaching through loss of Chl *a* in both *H. depressa* and *M. vertebralis*. A moderately significant correlation was found between reduced *F_v_*/*F_m_* and loss of Chl *a*, linking photodamage to bleaching. As the observed combined effects demonstrate a high level of agreement with the predicted combined effects as calculated through the combined effect model of Independent Action (IA), the experimental data are indicative of response additivity for this combination of stressors.

### Temperature

For the species evaluated here, elevated temperatures had an equivalent inhibitory effect on *ΔF*/*F′_m_* and *F_v_*/*F_m_* of symbionts *in hospite*, with the photosystem of diatom-bearing *H. depressa* and *A. quoyi* proving most sensitive. In these species, 96 hour exposures to temperatures of 32°C caused a considerable drop in *ΔF*/*F′_m_* and *F_v_*/*F_m_*, while 96 hours at 34°C proved lethal. Temperature-induced photodamage in *H. depressa* and *A. quoyi* as reported in this study was less extensive than described in a recent heat stress study on related diatom-bearing foraminifera from the Great Barrier Reef [62]. In that study, *F_v_*/*F_m_* in *H. depressa* was reduced by 25–45% after 6 days incubations at 32°C (as opposed to 10–20% reduction after 96 hours here), which is a further indication that photodamage caused by heat stress is likely to increase over time as demonstrated here (Fig. 4C). *Calcarina mayorii*, the other species tested hosting diatom symbionts, and *M. vertebralis* bearing dinoflagellates, were equally affected by temperature and somewhat less sensitive than *H. depressa* and *A. quoyi*, with only 96 hours at temperatures over 32°C affecting *ΔF*/*F′_m_* and *F_v_*/*F_m_* (Figs. 1 and 3). A recent study observed ∼15% inhibition *ΔF*/*F′_m_* in *M. vertebralis* when specimens were incubated at 32°C for 4 days, however higher light intensities were used [39]. Depth distributions of foraminifera vary greatly and are determined by temperature, light attenuation, water movement and substrate [68], [69]. On the GBR localities where experimental foraminifers were collected, *H. depressa* and *A. quoyi* typically exist in more shaded environments at depths between 9–15 m, often hidden deep within coral rubble or macroalgae and are therefore adapted to low irradiance and stable temperatures. *Calcarina mayorii* and *M. vertebralis* on the other hand, were collected at less than 7 m depth and are often found in shallower waters (or even on the reef flat) and will therefore be subject to greater temperature fluctuations, wave energy and higher solar irradiance. The dissimilar habitat types and associated adaptive ecology may partly explain the observed differences in sensitivity between species and related symbiont types. In accordance with results obtained in current study, Negri and colleagues [46] recently reported results where 10–15% inhibition of *ΔF*/*F′_m_* and *F_v_*/*F_m_* was observed in symbiotic dinoflagellates hosted by the branching coral *Acropora millepora* after 7 days incubation at 32°C, albeit at much higher levels of irradiance. At higher light intensities, the effects of heat stress may be intensified as absorbed excitation energy that cannot be transferred in photochemical pathways is instead passed on to form additional reactive oxygen [70]. Eventually, photo-oxidative stress renders symbiotic microalgae inefficient, potentially triggering a loss of symbionts (bleaching), possibly by host digestion [31], [71] or expulsion of the symbionts, a process frequently observed in hard corals [12], [72]. The fact that chronic photodamage was observed in this study under very low light intensities, further supports the suggestions that foraminifera are vulnerable to overexcitation and that irradiance intensity is an important limiting factor for the distribution and survival of species [42], [68], [73]–[75].

### Diuron

Exposure to 1 and 3 µg L^−1^ diuron for 96 hours significantly affected *ΔF*/*F′_m_* but the impact on *F_v_*/*F_m_* was less pronounced. Both ecotypes of *M. vertebralis*, hosting dinoflagellate symbionts, were slightly more sensitive to the negative effects of diuron than the species hosting diatoms, of which *H. depressa* was most vulnerable. Diuron-induced inhibition of *ΔF*/*F′_m_* after 96 hours as evaluated in this study was more severe than observed in a recent study [42]. However, that study used a lower experimental irradiance intensity (5 versus 10 µmol quanta m^−2^s^−1^ PAR) to examine a more acute effect (over 48 h). Previously we reported 10–25% inhibition *ΔF*/*F′_m_* in symbiotic diatoms after 48 hours incubation, against 20–35% inhibition observed in this study and this trend was similar for symbiotic dinoflagellates. Results obtained here indicate that maximum inhibition of *ΔF*/*F′_m_* in response to diuron is reached after 24 hours (Fig. 4), thus implying that different experimental irradiance intensities are primarily responsible for observed differences between the studies. The insensitivity of the red algae in this study can be explained by the fact that red algae can balance the excitation energy distribution between PSI and PSII, restricting herbicide effects and oxidative stress [76], limiting the usefulness of the saturation pulse method to assess the photosystem of red algae.

### Combined effects

Two stressors are considered biologically independent when the qualitative nature of the mechanism of action of one is not affected by the presence or absence of the other [77]. Moreover, the assessment of potential interaction can be unambiguously made, when taking this explicit non-interaction consideration as a reference model for evaluating observed combination effects. The combined effect model of IA used here revealed very consistent results across species with only minor diversions from the response additive model. The high level of agreement between the observed and predicted (IA) response demonstrates that the underlying simplistic mixture theory for this combination of stressors is valid and provides a useful tool for predictive modeling. ANOVA demonstrated significant individual effects of herbicide exposure and elevated temperature, with the only statistical interaction observed for inhibition *ΔF*/*F′_m_* in *M. vertebralis* (Table 2). In that species deviations from the predicted IA model indicated the response to these combined pressures to be sub-additive. Although the results reveal obvious differences in the sensitivity of a variety of symbiotic partnerships to the stressors tested, the experimental data are clearly consistent with IA, thus providing evidence that the risk from this combination of stressors is greater than from individual components.

### Symbiont type and shell ultrastructure

Similar to corals, foraminifera have been reported to host a wide variety of dinoflagellate clades of the genus *Symbiodinium* [78], [79] and these different clades may confer different stress tolerance characteristics, as has been reported for symbiotic dinoflagellates in corals [25], [26]. Cantin and colleagues [23] demonstrated that reduced photosynthetic output limits the translocation of carbohydrates from symbiont to host and that this effect was dependent on symbiont type. Reduced energy acquisition could decrease overall fitness and resilience of the host animal to further stressors. The results obtained here suggest that foraminifera hosting diatoms are more vulnerable to temperature stress and species hosting dinoflagellates more vulnerable to the effects of herbicides. Recently, we suggested a species' ultrastructure may influence diuron biokinetics as we observed delayed uptake and effect in porcelaneous (imperforate) species as opposed to hyaline (perforate) species [42]. Despite an equal sensitivity to the effects of elevated temperature, the current study revealed a slightly greater sensitivity to diuron in *H. depressa* when compared with co-existing *A. quoyi* (hyaline versus porcelaneous, respectively; both hosting diatoms). *Marginopora vertebralis* (porcelaneous; dinoflagellates) was more heavily affected by diuron than either hyaline or porcelaneous diatom-bearing species, while at the same time less vulnerable to elevated temperature as assessed by PAM-fluorometry. Following our results, improving water quality (by reducing herbicide levels) will have the greatest effect for the diatom-bearing species *H. depressa* and *A. quoyi*, as these species are most likely to suffer the effects from elevated SSTs. The symbiotic dinoflagellates tested here may be less vulnerable to thermal pressure than diatoms, but suffer more stress from herbicides alone. For short-term exposures this may not be a problem, but it remains unclear how foraminifera respond to longer-term exposures of physical or chemical stress.

### Ecological effects

Both *ΔF*/*F′_m_* and *F_v_*/*F_m_* have been found to rapidly recover after the responsible stress factor had been removed in various corals [80], [81] and benthic foraminifera [42]. However, sustained reductions in *F_v_*/*F_m_* can lead to reduced growth and loss of symbionts or photosynthetic pigmentation, as has been observed in corals [82] and now foraminifera. Corals exposed to diuron for 2–3 months exhibited decreased lipid content, bleaching, curbed reproductive success as well as colony mortality [82]. The correlation between reduced *F_v_*/*F_m_* and bleaching has often been observed in corals as a consequence of environmental stressors such as high temperatures [83], high irradiance intensities [70], reduced salinity [84], herbicides [85] or combinations thereof [46]. While tissue bleaching in corals is considered a sub-lethal stress response and may be reversed, partial or whole-colony mortality often results [5]. In foraminifera, evidence exists of bleached populations recovering in late summer and fall or over multiple years following mass bleaching events [32], [86]. On the other hand, environmental stress can induce abnormal reproduction and cellular damage [71], [87], affect structural integrity and immune response [28], [88], potentially leaving species vulnerable to disease, predation and further stressors. Moreover, previous studies have demonstrated how assemblages can shift from being dominated by large, symbiont-bearing foraminifers to smaller, herbivorous or detrivorous species under the influence of environmental stress [28], [89].

### Implications

Whereas thermal stress has been proposed as the main physiological driver behind mass coral bleaching events [2], [90], evidence is emerging that water quality may have a strong influence on the sensitivity of reef species to physical stressors as elevated SSTs and ocean acidification [15], [39], [46], [47], [91], [92]. In Queensland and other tropical environments, high summer temperatures often coincide with monsoonal rainfall events, responsible for the delivery of the highest annual loads of fresh water, sediments, nutrients and associated pesticides onto nearshore areas of the GBR [19], [93]. Thus it is likely that inshore primary producers such as corals, seagrasses and foraminifera are simultaneously exposed to chemical and physical stressors. Our results indicate that minimizing pollution can reduce total pressure phototrophic organisms experience under conditions of thermal stress. Water quality guidelines for contaminants as well as laboratory experiments directed at evaluating temperature stress thresholds often do not take into consideration the highly likely scenario that sensitive organisms will be exposed to combined and/or cumulative stressors, potentially underestimating the true extent of environmental pressure. These pressures on reef ecosystems are likely to increase further as a result of expanding coastal development, population growth and climate change. While limiting the effects of climate change is a global challenge, policies minimizing the effects of pollution can contribute towards the survival and sustainable exploitation of our marine resources. Restricting the inflow of suspended sediments, nutrients and chemical contaminants represents a practical local strategy to protect our reef ecosystems in a changing environment [15], [94], [95].
